# The Developmental Transcriptome of the Mosquito *Aedes aegypti*, an Invasive Species and Major Arbovirus Vector

**DOI:** 10.1534/g3.113.006742

**Published:** 2013-09-01

**Authors:** Omar S. Akbari, Igor Antoshechkin, Henry Amrhein, Brian Williams, Race Diloreto, Jeremy Sandler, Bruce A. Hay

**Affiliations:** Division of Biology, MC 156-29, California Institute of Technology, Pasadena, California 91125

**Keywords:** *Aedes aegypti*, dengue fever, yellow fever, chikungunya, malaria, population replacement, transcriptomes, Medea, gene drive

## Abstract

Mosquitoes are vectors of a number of important human and animal diseases. The development of novel vector control strategies requires a thorough understanding of mosquito biology. To facilitate this, we used RNA-seq to identify novel genes and provide the first high-resolution view of the transcriptome throughout development and in response to blood feeding in a mosquito vector of human disease, *Aedes aegypti*, the primary vector for Dengue and yellow fever. We characterized mRNA expression at 34 distinct time points throughout *Aedes* development, including adult somatic and germline tissues, by using polyA+ RNA-seq. We identify a total of 14,238 novel new transcribed regions corresponding to 12,597 new loci, as well as many novel transcript isoforms of previously annotated genes. Altogether these results increase the annotated fraction of the transcribed genome into long polyA+ RNAs by more than twofold. We also identified a number of patterns of shared gene expression, as well as genes and/or exons expressed sex-specifically or sex-differentially. Expression profiles of small RNAs in ovaries, early embryos, testes, and adult male and female somatic tissues also were determined, resulting in the identification of 38 new *Aedes*-specific miRNAs, and ~291,000 small RNA new transcribed regions, many of which are likely to be endogenous small-interfering RNAs and Piwi-interacting RNAs. Genes of potential interest for transgene-based vector control strategies also are highlighted. Our data have been incorporated into a user-friendly genome browser located at www.Aedes.caltech.edu, with relevant links to Vectorbase (www.vectorbase.org)

*Aedes aegypti* is the principal vector for the flaviviruses yellow fever and dengue fever (Barrett and Higgs 2007; Halstead 2008) and is also responsible for several recent outbreaks of the alphavirus chikengunya (Ligon 2006). Approximately 2.5 billion people are at risk for dengue, with 50−100 million cases and ~25,000 deaths each year (Guzman and Isturiz 2010). The range of *A. aegypti* is expanding through tropical and subtropical zones worldwide, and the occurrence of Dengue fever has closely followed the expansion of the mosquitoes (Guzman *et al.* 2010; Guzman and Isturiz 2010; Lounibos 2002). No vaccine is available, leaving vector control the only option for prevention. The emergence and spread of insecticide resistance poses a threat to the sustainability of these efforts (Hemingway *et al.* 2002). Vaccines are available for yellow fever, but there are still ~200,000 cases each year, resulting in ~30,000 deaths (Barrett and Higgs 2007). Many *Aedes aegypti* strains also are susceptible to infection with West Nile virus (Vanlandingham *et al.* 2007), the avian malaria parasite *Plasmodium gallinaceum* (James 2002), and filariatic nematodes (Erickson *et al.* 2009), making this organism an important tool for the investigation of multiple mosquito-pathogen interactions. *Aedes aegypti* is also a model organism for mosquito biology. It is easy to transition from field to laboratory culture, fertilized eggs can be stored in a diapause state for many months, and it is straightforward to transform with the use of transposons and site-directed integration systems (Nimmo *et al.* 2006; Terenius *et al.* 2008). Much of what we know about mosquito genetics, biochemistry and behavior comes from study of this species (Clements 1999; Clemons *et al.* 2010; Severson and Behura 2012; Vanlandingham *et al.* 2007).

To understand mosquito development, how the insect adapts to specific environments, acquires resistance to insecticides, and responds to infection by pathogens, the full genomic complement of genes, their structures, and the patterns of gene expression associated with these activities is needed. This, in conjunction with the ability to manipulate gene expression, provides a platform from which to generate and test hypotheses about the functions of specific genes. The *cis*-acting elements that drive gene expression in specific patterns—the identification of which is facilitated through detailed transcriptional profiling of many states and the identification of transcription start sites—also are needed to develop novel forms of transgenic-based vector control that involve population suppression or replacement of wild populations with individuals refractory to disease transmission (Alphey *et al.* 2008; Burt 2003; Catteruccia *et al.* 2009; Chen *et al.* 2007; Davis *et al.* 2001; Hay *et al.* 2010; Sinkins and Gould 2006).

The *Aedes aegypti* genome project identified 15,419 gene models by using a large collection of expressed sequence tags and gene structure information from other diptera (Nene *et al.* 2007). These data have provided the basis for a number of transcriptional profiling experiments that use oligonucleotide probes on arrays (Behura *et al.* 2011; Caragata *et al.* 2011; Colpitts *et al.* 2011; Dissanayake *et al.* 2010; Erickson *et al.* 2009; Ptitsyn *et al.* 2011; Sim and Dimopoulos 2010; Souza-Neto *et al.* 2009; Xi *et al.* 2008; Zhu *et al.* 2010; Zou *et al.* 2011). These have provided information on gene expression in response to a variety of stimuli and in some cases throughout portions of development. However, transcriptional profiling via the use of microarrays is limited in that arrays often contain probes for only a subset of known genes, they do not allow for the identification and characterization of new genes, and their use suffers from various technical biases such as nonspecific hybridization and insufficient signal for genes expressed at low levels. In contrast, transcriptome information generated by the use of massively parallel cDNA sequencing (RNA-seq) provides absolute measures of gene expression and can quantify the levels of known and unknown genes, facilitating annotation of the genome while identifying patterns of gene expression (Mortazavi *et al.* 2008). RNA-seq can also be used to study differential splicing and locate precise transcription start and stop sites, all at single-base resolution (Ozsolak and Milos 2011). Several recent studies have used RNA-seq to characterize *A. aegypti* polyA+ mRNA or microRNA transcriptomes in specific contexts, and novel genes and patterns of gene expression have been identified (Biedler *et al.* 2012; Biedler and Tu 2010; Bonizzoni *et al.* 2011, 2012a,b; David *et al.* 2010; Gibbons *et al.* 2009; Hess *et al.* 2011; Neira-Oviedo *et al.* 2011; Paris *et al.* 2012; Rinker *et al.* 2013; Surget-Groba and Montoya-Burgos 2010). However, these studies focused on characterization of only a few life cycle stages, leaving much of the transcriptome yet to be explored. No comprehensive transcriptome encompassing the development of an arthropod vector of disease has been reported by the use of array-based transcriptional profiling or RNAseq, although a detailed array-based analysis of gene expression during *Anopheles gambiae* embryonic development and studies of sex- and tissue-specific gene expression have been reported (Baker *et al.* 2011; Goltsev *et al.* 2009; Magnusson *et al.* 2011).

Here, we provide a comprehensive analysis of the *A. aegypti* transcriptome throughout development, sequenced at unprecedented depth using RNA-seq. These data have been incorporated into a genome browser located at aedes.caltech.edu, with links to Vectorbase (www.vectorbase.org) to provide easy access to genomic information.

## Materials and Methods

### Mosquitoes and sample time points

Mosquitoes used for RNA extraction were from the *A. aegypti* Liverpool strain, which originated from West Africa, and was used to produce the *A. aegypti* reference genome (Nene *et al.* 2007). Mosquitoes were kept in incubators with a relative humidity of 70–80%, maintained at 28º, and with a 12-hr/12-hr light/dark cycle. Larvae were fed with ground fish food (TetraMin Tropical Flakes, Tetra Werke, Melle, Germany) and sex-separated as pupae. Adults were maintained and fed with an aqueous solution of 30% sucrose. Females were blood-fed 3−5 d after eclosion on anesthetized mice and then returned to normal mosquito-rearing conditions during sample collections. All animals were treated according to the Guide for the Care and Use of Laboratory Animals as recommended by the National Institutes of Health.

### Total RNA isolation

Samples were flash-frozen at specific time points, and total RNA was extracted by use of the Ambion mirVana mRNA isolation kit (Ambion/Applied Biosystems, Austin, TX). All sample collections were staged in the incubator at a relative humidity of 70–80%, 28º with a 12-hr/12-hr light cycle until the desired time point was reached. Samples were then immediately flash frozen. The adult NBF carcass was processed at 3 d after eclosion, and the adult male carcass and testes were processed at 4 d after eclosion. After extraction, RNA was treated with Ambion Turbo DNase (Ambion/Applied Biosystems, Austin, TX). The quality of RNA was assessed using the Bioanalyzer 2100 (Aglient Technologies, Santa Clara, CA) and the NanoDrop 1000 UV-VIS spectrophotometer (NanoDrop Technologies/Thermo Scientific, Wilmington, DE). RNA was then prepared for sequencing using the Illumina mRNA-Seq Sample Preparation Kit (Illumina San Diego, CA).

### Small RNA extraction, cloning, and sequencing

Total RNA was extracted from ovaries of NBF females, and PBM, at 24-, 48-, and 72-hr time points. RNA was also extracted from 0- to 2-hr and 2- to 4=hr embryos, female carcasses 72 hr PBM, male carcasses lacking testes and AG, and testes plus AG. Twenty micrograms of total RNA from each sample was size fractionated on 15% TBE-Urea polyacrylamide gels. For all ovary and embryo time points small RNAs between 18−26 nt and 26−32 nt in length were excised and sequenced separately. For the male and female carcasses and testes and AG samples small RNAs between 18−32 nt were sequenced as a single sample. Ethanol precipitated RNA was ligated to a HPLC purified 3′ linker using T4 RNA ligase (Ambion/Applied Biosystems, Austin, TX). Ligation products were purified on a 15% TBE-Urea polyacrylamide gel and recovered by high-salt elution. A high-performance liquid chromatography−purified 5′RNA linker was ligated to the product using T4-RNA ligase, and the product was purified on a 15% TBE-Urea polyacrylamide gel and recovered as described previously. Reverse transcription was performed using SSIII (Invitrogen, Carlsbad, CA), and cDNA was amplified using Phusion polymerase (Finnzymes Oy, Espoo, Finland). Amplified cDNA libraries were purified on a 2% agarose gel and sequenced using the Illumina Genome Analyzer II system. Linker and primer sequences are provided in supplementary Supporting Information, Table S23.

### Poly(A+) read alignment and quantification

Poly(A+) transcriptome reads were processed and aligned to a reference index generated for the A. aegypti genome (AaegL1, obtained from www.vectorbase.org), using TopHat v1.2.0 (Trapnell *et al.* 2009). Reads were aligned using both default parameters allowing up to 40 alignments per read with a maximum 2-bp mismatch and also using unique mapping allowing only one alignment per read with a maximum 2-bp mismatch (both datasets are provided). The aligned read files were processed by Cufflinks v0.9.3 (Trapnell *et al.* 2010). Cufflinks uses the normalized RNA-Seq fragment counts to measure the relative abundances of transcripts. The unit of measurement is fragments per kilobase of exon per million fragments mapped (FPKM).

### Small RNA read alignment and quantification

The 5′ and 3′ adapter sequences for the small RNA reads were removed using custom Perl scripts that required a minimal match to the adapter sequence of 6 bp and a minimal size of 18 bp and maximum size of 32 bp (sequences for the adapter sequences supplied in Table S23). The trimmed sequences were aligned to the *A. aegypti* reference genome using bowtie v0.12.7, allowing no mismatches and a maximum of 10 alignments/read. We determined small RNA abundance using custom in house Perl scripts in which we quantified the read count per million of mapped reads (RPM) for each genomic position for all nine libraries. To identify novel and non-annotated miRNA orthologs, we then sorted for the most abundant (>50 read counts) mapped reads in the size range of 19−24 bp across all nine libraries and filtered out all the annotated miRNAs. We isolated ~60-bp flanking each mature sequence and tested its ability to fold into a miRNA stem loop structure using Mfold default settings (http://mfold.rna.albany.edu/?q=DINAMelt/Quickfold).

### TE expression

A bowtie index was created from all annotated TE in A. aegypti (extracted from http://tefam.biochem.vt.edu/tefam/) and both the long poly(A+) transcriptome reads and the adapter trimmed small RNA reads were aligned to this index using bowtie. Expression scores (RPKM) for each TE element were calculated using custom scripts in which each score was divided by the total length (in kb) of each annotated TE and this value was normalized to the total mapped reads (in millions) in that sample (instead of normalizing to just the reads which map to TE elements). The normalized expression values for each TE in each class were summed to illustrate the TE class expression values.

### Discovery of new isoforms and newly transcribed regions (NTRs)

Reads from four sex-specific paired end sequenced libraries and 42 single-read sequenced libraries were aligned to the Aedes genome using tophat (v1.3.3) allowing the discovery of novel junctions. *De novo* transcriptome assembly was carried out for each mapping file separately using cufflinks (v1.3.0). GTF files produced by cufflinks runs were merged with the cuffmerge utility of the cufflinks package. Cuffmerge was also used to compare *de novo* transcriptomes to existing AAEL transcripts. The combined GTF file was parsed to identify new isoforms of AAEL genes (class code “j”) and NTRs (class code “u”). Nucleotide sequences of newly identified transcribed regions were extracted and searched against the nonredundant protein database downloaded from NCBI (on 7/23/2012) using NCBI BLAST+ (Camacho *et al.* 2009). Conserved protein domains and associated GO terms were identified using stand alone InterProScan package v4.9 and the database release 38.0 (June 2012) (Zdobnov and Apweiler 2001).

### Clustering and Gene Ontology (GO) analysis

Cufflinks-produced FPKM values for 42 RNA-seq libraries were clustered using Mfuzz R software package (Kumar and Futschik 2007). Mfuzz uses fuzzy c-means algorithm to perform soft clustering, which allows cluster overlap and has been demonstrated to perform favorably on gene expression data. The resulting clusters were analyzed for overrepresentation of GO terms using a hypergeometric test implemented using the GOstats R software package (Falcon and Gentleman 2007). GO annotations for known genes were downloaded from VectorBase and merged with GO terms produced by the InterProScan analysis of novel genes. Hypergeometric tests were performed separately for biological process, molecular function, and cellular component ontologies.

### DEXSeq differential exon usage analysis

Differential exon usage was analyzed using DEXSeq (ver. 1.1.7) R package (http://bioconductor.org/packages/release/bioc/html/DEXSeq.html) according to the package manual. In summary, annotation file in GTF format containing annotated VectorBase AAEL gene models and cufflinks-identified NTRs (AAEL-NIPS and NTRs) was processed with prepare_annotation.py script provided with DEXSeq package to define nonoverlapping exonic parts. Exon read counts for four sex-specific libraries (male carcass, male testes, blood-fed female carcass, blood-fed ovaries) sequenced as paired end 76 nt and single read 38 nt were generated from tophat-produced mapping files in BAM format using dexseq_count.py script. Counts from libraries for each sex were combined to generate two male and two female-specific counts (one for each run type). The count data were imported into the DEXSeq framework and differential exon usage was assessed using make CompleteDEUAnalysis function with default parameters. The analysis identified 2,468 differentially used exonic parts originating from 1278 loci at FDR of 0.05. HTML reports and graphics were generated using DEXSeqHTML function.

## Results

### Strategy for the characterization of *A. aegypti* transcriptome

To establish a comprehensive view of gene expression dynamics throughout *A. aegypti* development we conducted poly A(+) RNA sequencing (poly A(+) RNA-seq) using RNA isolated from 42 samples representing 34 distinct stages of development from the *A. aegypti* Liverpool (*i.e.*, LVP) strain (Nene *et al.* 2007; Table S1). These time points incorporated 26 whole animal and 16 tissue/carcass samples. For embryogenesis we collected 20 samples; the first three time points, 0−2 hr, 2−4 hr, and 4−8 hr embryos, capture the maternal-zygotic transition at 2-hr intervals, whereas 17 additional samples collected through 76 hr, at 4-hr intervals, capture the rest of embryogenesis. Samples also were collected from each of the four larval instars and sex-separated male and female pupae. Sixteen additional samples were collected from adults. These include dissected whole ovaries from nonblood-fed females (NBF) and from females at 12 hr, 24 hr, 36 hr, 48 hr, 60 hr, and 72 hr postblood-meal (PBM); carcass samples (whole female bodies lacking ovaries) also were collected from these same time points. These samples cover ovarian and follicle development from previtellogenic “resting stage” NBF ovaries through the completion of oogenesis at 72-hr PBM. We also isolated samples from adult male testes and accessory glands (AG) as a single sample and male carcasses (lacking testes and AG) at 4 d after eclosion. Table S1, Table S2, and Table S3 provide a summary of the complete polyA+ transcriptome.

Samples for sequencing of small RNAs were prepared from nine stages designed to characterize small RNAs through several major developmental transitions and in germline and somatic tissues. Samples from NBF ovaries, and ovaries from blood-fed females at 24 hr, 48 hr, and 72 hr span oogenesis and should be enriched in small RNAs present in the adult female germline and surrounding somatic support cells. Small RNAs from 0−2 hr and 2−4 hr embryos should consist of maternally expressed RNAs deposited into the egg but not include those present in ovarian support cells during oogenesis. Samples were also prepared from adult male carcasses, testes + AG, and adult female carcass 72 hr PBM, providing additional information on small RNAs that are expressed specifically in the germline, soma, or sex specifically.

To achieve single nucleotide resolution and facilitate the discovery of non-annotated genes, we used the Illumina Genome Analyzer II sequencing platform to sequence the aforementioned samples, producing a combination of single 38 nt and paired-end 76-bp reads. In total we generated 1.2 billion single 38 nt and paired 76-nt raw reads corresponding to total sequence output of 46 GB, with 89.45% of the reads (1.1 billion reads, 41.8GB of sequence) aligning to the *Aedes aegypti* genome (Table S2).

### Discovery of NTRs

The current assembly of the *A. aegypti* genome (AaegL1, released September 2009) contains 4758 supercontigs and is 1,310,090,344 bp in size, 4.7 times larger than the genome of the malaria mosquito *Anopheles gambiae*. The existing genomic annotation, which contains 17,346 genes (hereafter referred to as AAEL genes) and 18,760 transcripts encoding 17,402 peptides, was used as a starting point for our analysis (Nene *et al.* 2007). Sequence reads from four sex-specific, paired-end–sequenced libraries, and all 42 single-end-sequenced libraries, were used to build *de novo* transcriptomes for each sample with the use of Cufflinks (Trapnell *et al.* 2010). Individual transcriptomes were merged to produce a combined gene set, which included 18,755 known transcripts, 20,198 novel isoform predictions (NIPs) of 6913 existing AAEL genes (hereafter referred to as AAEL-NIPs), as well as 14,238 NTRs that do not overlap AAEL transcription units or other annotated genome features potentially transcribed, such as transposable elements (TEs). These NTR transcripts are predicted to arise from 12,597 loci (File S1 and File S2). Leveraging paired end and splice junction information present in our data, the transcriptome assembly aggregated 2,676 AAEL genes into 1,231 independent loci (File S3). The new transcriptome assembly confirmed 47,841 of 48,089 junctions derived from previously annotated AAEL transcripts (99.48%) and defined 29,242 novel junctions, significantly increasing potential splicing complexity of *Aedes* transcriptome. A total of 23,419 (80%) of the newly discovered junctions belong to AAEL-NIPs and 5823 (20%) originate from NTR transcripts.

To put these findings in perspective, the AAEL transcriptome covers 26.01 MB of genomic sequence (1.99% of the assembled genome). The AAEL-NIPs increase the genomic sequence coverage to 37.20 MB, and NTRs add another 13.27 MB. Thus, the transcripts identified from our polyA+ RNA samples result in an increase of transcribed sequence from 26.01 MB to 49.99 MB, almost doubling the fraction of the genome transcribing long polyA+ RNAs.

To get a better understanding of what the NTRs encode, we conducted homology searches against the NCBI nonredundant protein database and identified significant hits for 5351 (37.58%) NTRs (Table S4). For 1707 (31.90% of hit-producing NTRs) NTRs, the best blast hits were against *A. aegypti* proteins, suggesting that they may represent paralogs of previously annotated AAEL genes. The next most commonly identified species was *Culex quinquefasciatus*, producing best hits for 1173 transcripts. *Anopheles gambiae*, *Anopheles darling*, and *Aedes albopictus* proteins were found to be most homologous to 528, 285, and 30 NTRs, respectively. Altogether, members of the mosquito family produced best hits for close to 70% of NTRs. Various members of the *Drosophila* genus produced best hits for additional 7.6% of transcripts, *Drosophila willistoni* being most frequently seen species with 147 hits. Of 8,886 NTRs lacking significant blastx hits, just 105 have open reading frames (ORFs) longer than 200 amino acids, and only 14 of these contain protein domains identifiable by interproscan (Table S5). These results suggest that the majority of NTRs with no significant protein homology represent either noncoding RNAs or novel short protein-coding transcripts specific to *Aedes*.

To further explore the possibility that NTRs without significant protein homology are noncoding we analyzed them using the program Coding Potential Calculator (Kong *et al.* 2007). Although Coding Potential Calculator has a low false-positive rate (1%) when tested against *Drosophila* modENCODE protein-coding transcripts (Abedini and Raleigh 2009), the same settings gave an unacceptably high-false positive rate (25%) when tested against AAEL genes known or thought to encode proteins. As an alternative, we identified all transcripts longer than 200 nt, with an FPKM of 10 or greater in at least one sample, and that lacked an ORF of 200 amino acids or longer (Young *et al.* 2012), which resulted in a list of 3070 candidate highly expressed noncoding RNAs (File S4 and Table S6). To visualize the major patterns of coregulated noncoding NTR expression we used a soft clustering algorithm (Kumar and Futschik 2007). This resulted in the identification of multiple developmental expression patterns: the ovary and early embryo (cluster 1); early embryo-specific (cluster 9); mid- and later embryogenesis (clusters 2 and 3); late embryogenesis (cluster 5); pupal stages (cluster 7); adult somatic tissues (cluster 10); adult ovary and early embryo (cluster 8); adult male germline (cluster 4); male germline and nonblood-fed ovary (cluster 6; Figure S1 and Table S7).

The large number of potentially noncoding NTRs expressed relatively specifically in the nonblood-fed ovary and/or testis + AG is striking (Figure S1, cluster 6 and cluster 4; Table S7). Roles for noncoding RNAs in insects are just beginning to be explored, but it is interesting to consider that important roles for noncoding RNAs in stem cells in mammalian systems have recently been described (Bertani *et al.* 2011; Loewer *et al.* 2010; Sheik Mohamed *et al.* 2010). The nonblood-fed ovary contains quiescent stem cell-like populations for both germline and somatic cells of developing egg follicles, which are activated to begin proliferation and differentiation in response to a blood meal. The testes also contain somatic and germline stem cells. It will be interesting to determine the consequences of knocking out the functions of some of these on early germline development.

### Global transcriptome dynamics

**Figure 1:**
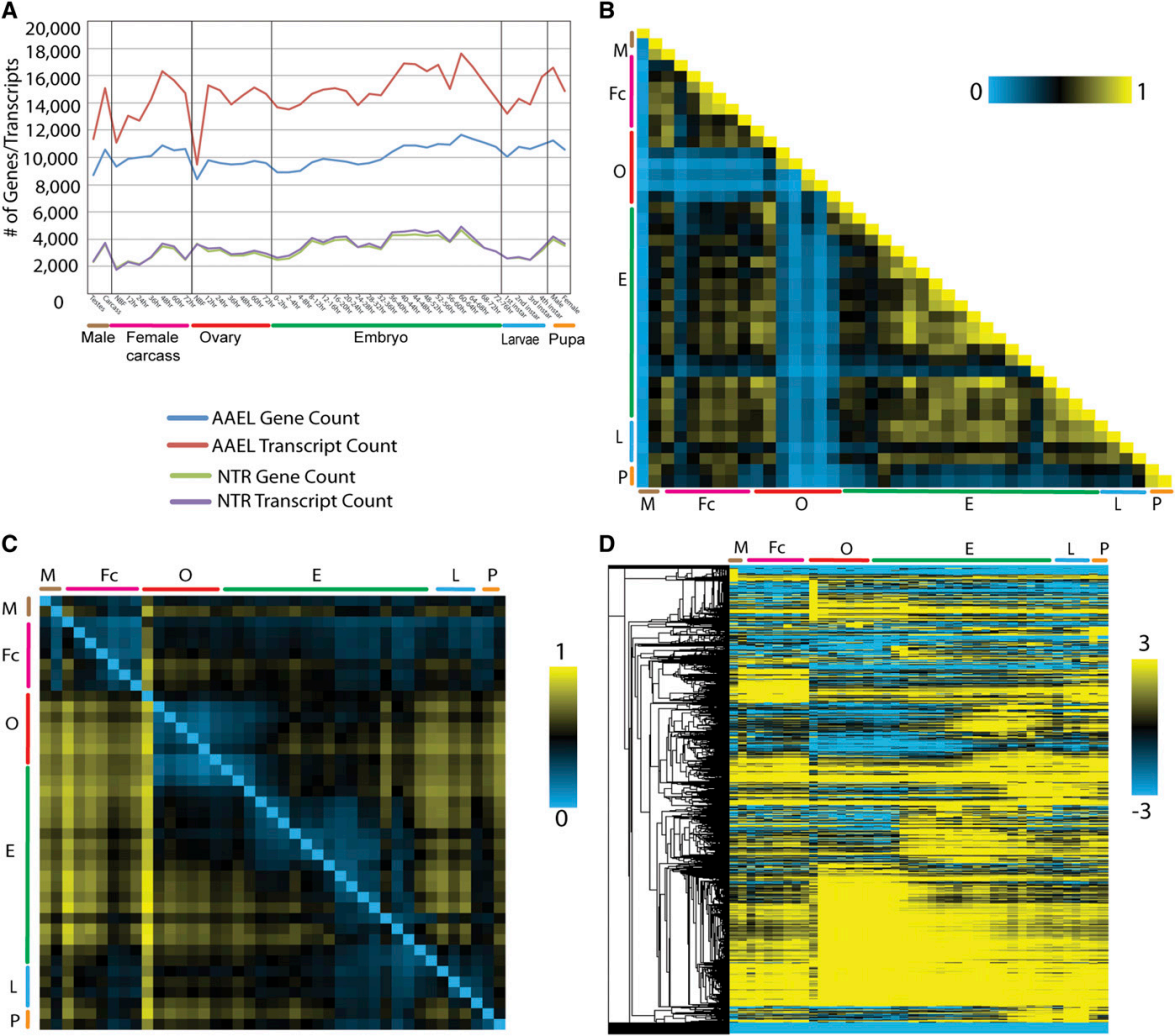
Global dynamics of gene expression. The number of expressed (FPKM > 1) AAEL genes (blue) and AAEL and AAEL-NIP transcripts (red) and NTR gene (green), and NTR transcripts (purple) were plotted across all 42 developmental time points (A). Correlation matrix of all 42 poly (A+) RNA seq time points throughout development for AAEL genes and NTRs. Each developmental stage is most highly correlated with its adjacent time point across all embryogenesis. A decrease in correlation is observable in the 36−48hr ovary and 60−72hr ovary, 52−56hr to 56−60hr embryo. The scale bar indicates the coefficient of variation value between samples 0−1 (B). The expression heat map indicates the number of AAEL genes and NTRs that are fivefold upregulated between each sample. The number of AAEL genes and NTRs that are 5 fold up-regulated can be determined by matching the criteria with respect to the sequence of the row tissue (left) to the column tissue (top). For example, there are 10,762 (yellow, highest number of expressed genes and this value is 1) genes and NTRs that have 5-fold more transcriptional activity in the 24hr BF ovary tissue (left) compared with the NBF ovary tissue (top). In addition, there are 4302 (0.399 value in chart) genes and NTRs (blue), which have 5-fold more transcriptional activity in the NBF ovary tissue (left) compared with the 24-hr BF ovary tissue (top). These two statements are mutually exclusive and therefore each cell represents a different set of genes (C). Hierarchical clustering heat map of AAEL genes and NTRs, illustrating the various patterns of gene expression across all developmental time points. Scale bar indicates the FPKM z scores (D). For A−D, The major developmental groups are indicated by color bars and are organized left to right, as follows: M (brown, male testes, male carcass), Fc (purple, NBF Female Carcass, and multiple time points PBM: 12hr, 24hr, 36hr, 48hr, 60hr, and 72hr), O (red, NBF ovaries, and multiple ovarian time points PBM: 12hr, 24hr, 36hr, 48hr, 60hr and 72hr), E (green, embryo, 0-2hr, 2-4hr, 4-8hr, 8-12hr, 12-16hr, 16-20hr, 20-24hr, 24-28hr, 28-32hr, 32-36hr, 36-40hr, 40-44hr, 44-48hr, 48-52hr, 52-56hr, 56-60hr, 60-64hr, 64-68hr, 68-72hr and 72-76hr embryos), L (light blue, larvae, 1^st^, 2^nd^, 3^rd^ and 4^th^ instar larvae stages), and P (light orange, male and female pupae).

To examine the dynamics of gene expression, we quantified expression changes of AAEL, NTR, and AAEL-NIP gene/transcript models across all developmental samples (Figure 1A, Table S8, and Table S9). NBF ovaries express the lowest number of genes (8400) with close to 1.1 isoform per gene. An increase in isoform complexity, which increases to 1.6 transcripts per gene, the highest in the dataset, is seen upon a blood meal. The number of expressed genes and isoforms gradually rises through embryogenesis, reaching a peak at 60 hr and decreasing afterward. Analysis of pair-wise correlations in expression levels of AAEL and NTR genes revealed that almost every developmental stage is most highly correlated with its adjacent stage, particularly during embryogenesis (Figure 1B). Notable exceptions to this trend occur between 36−48 hr and 60−72 hr ovaries, and between the 52−56 hr and 56−60 hr embryo stages, suggesting that these represent important points at which developmental and/or physiological transitions occur.

To identify general trends in expression profiles we compared the transcription count for every sample with every other sample, looking specifically at NTRs and AAEL genes (Figure 1C). Two of the most prominent features to emerge from this analysis are the unique transcriptional signature of the nonblood-fed ovary, which presumably reflects its biologically unique state as a repository of quiescent somatic and germline stem cells, and the large number of genes whose expression is up-regulated at least fivefold in the ovary PBM, reflecting the maternal synthesis of products required for oocyte formation. Large changes in gene expression during a gonotrophic cycle have also been noted in *Anopheles gambiae* (Dana *et al.* 2005; Marinotti *et al.* 2006).

**Figure 2:**
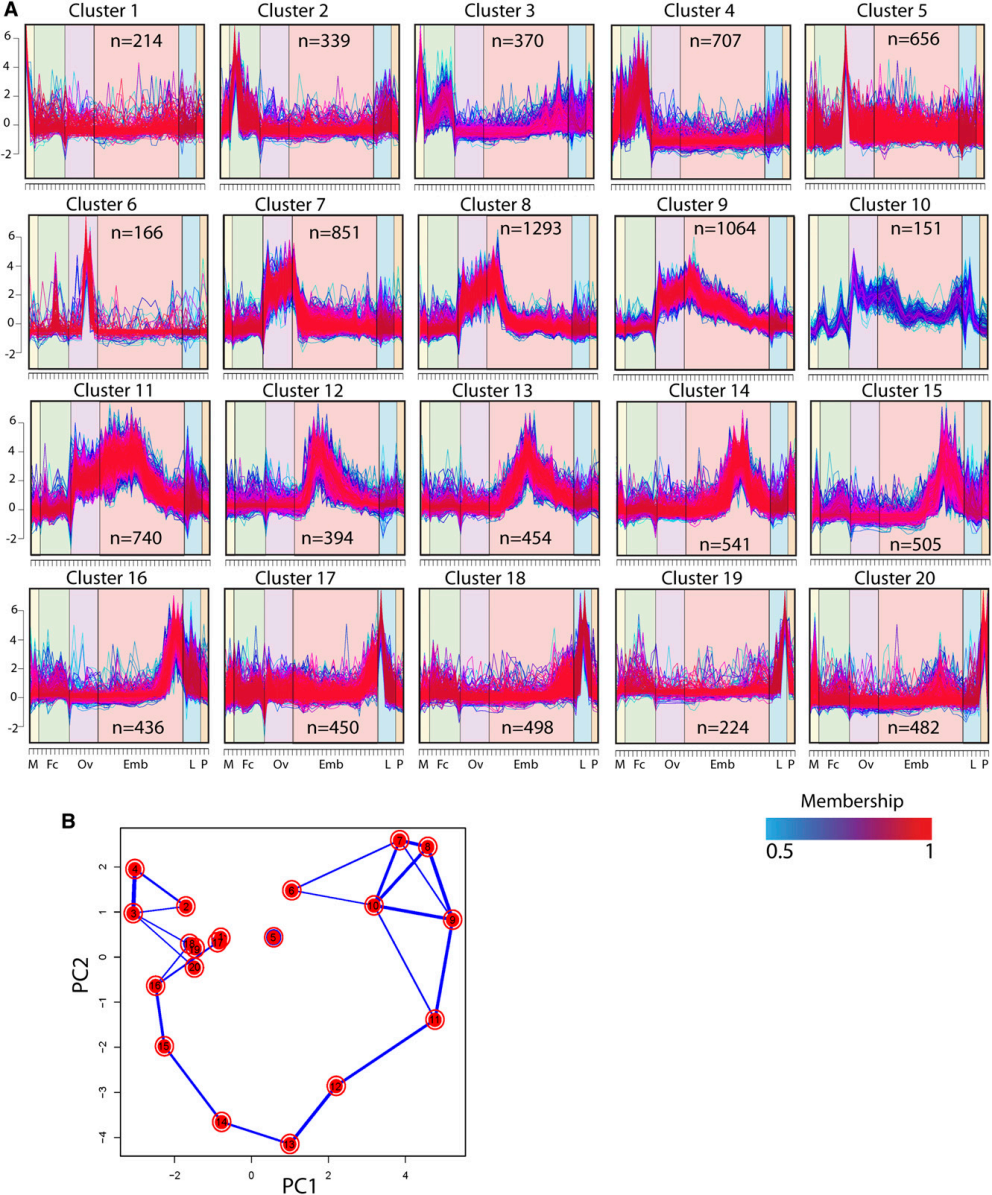
Soft clustering, principal component analysis, and totals. Twenty AAEL gene expression profile clusters were identified through soft clustering. Each gene is assigned a line color corresponding to its membership value, with red (1) indicating high association. The major developmental groups are indicated by symbols on the X axis, and are organized as in Figure 1, B−D (A). Principal component analysis shows relationships between the 20 clusters, with thickness of the blue lines between any two clusters reflecting the fraction of genes that are shared (B, thickness of blue lines). n, the number of genes in each cluster.

A global view of annotated AAEL and NTR gene expression is shown in the hierarchical clustering heat map in Figure 1D. To better visualize the major patterns of co-regulated gene expression for the AAEL genes we used a soft clustering algorithm, and identified 20 distinct patterns that included from 151 to 1293 genes (Figure 2, Table S10, and Table S30). Many of these patterns correspond to specific developmental stages and transitions. For instance cluster 5 includes genes specifically expressed in the NBF ovary. It is highly enriched in genes involved in translation, including 92 tRNA and 12 rRNA genes. Seventy-one percent (n = 47) of the annotated snRNAs present in the *Aedes* genome also are found in this cluster, suggesting that mRNA splicing and maturation are also particularly important during this stage. Genes involved in mitochondrial biogenesis and RNA polymerase function are also over represented. Perhaps most strikingly, cluster 5 also expresses a large number of putative olfactory and gustatory receptors, raising the interesting possibility that environmental cues sensed by the ovary PBM may be important in initiating ovarian development. The dramatic switch from low to high transcriptome complexity following this life stage (Figure 1A), and the accumulation of genes required for response to stimuli and RNA processing, suggest that NBF ovary is poised for rapid transcriptional response and growth that occurs PBM. Cluster 6 includes genes predominantly expressed somewhat later in oogenesis. These include many genes involved in polysaccharide or carbohydrate binding, as well as peroxidases and oxireductases, which are likely involved in chorion and vitelline membrane biosynthesis. Interestingly, cluster 6 is also highly enriched in odorant-binding proteins. Other genes that are induced in response to a blood meal are grouped in clusters 7, 8, 9, and 11. Cluster 7 includes strictly ovary-specific genes, whereas the others include genes whose expression initiates PBM and extends into embryogenesis. Cluster 8 in particular, the largest cluster produced, includes maternally expressed genes deposited into the embryo, and is enriched in genes involved in response to stimuli, signal transduction, and protein modification among others. Clusters 12 through 16 identify genes with increased expression during early, mid and late embryogenesis; clusters 17, 18, and 19 include larvae-specific genes, while cluster 20 contains genes preferentially expressed in pupae.

### Sex-specific gene expression and splicing

Males and females differ in many morphologic, behavioral, and physiologic traits, largely caused by differences in gene expression. To begin to study these differences we compared transcriptomes from male and female samples. Figure S2, A−E shows plots of expression level and sex bias for pupae, whole adults, and carcass and germline. We observed male biased AAEL and NTR gene expression when comparing male carcass with NBF-female carcass (8196 genes and NTRs, 23% of all detected genes and NTRs expressed), male pupae to female pupae (6265 genes and NTRs, 17.5%), male carcass to 72hr-BF-female carcass (3420 genes and NTRs, 9.5%), and male testes and AG to NBF female ovaries (218 genes and NTRs, 0.6%). In contrast, a strong female bias was observed in comparisons between 72-hr BF ovaries and testes plus AG (6165, 17.2%), presumably reflecting the large number and diversity of transcripts deposited in the mature oocyte. An overall male bias in gene expression was recently reported in Drosophila when comparing whole adults, and was proposed to be due to the transcriptional complexity of the testes (Graveley *et al.* 2011). Our results suggest that male biased gene expression also occurs in somatic tissues.

A number of AAEL genes and NTRs were identified from our sex-specific samples as being expressed at levels >20× in one sex or the other: 234 in males (63% NTRs, 37% AAEL) and 3783 in females (43% NTRs, 57% AAEL; Table S28 and Table S29). When the sex-specific expression criteria were tightened to include only those genes expressed strictly sex-specifically (0 reads in the other sex), a much smaller number of genes are identified: 81 in males and 924 in females (Table S11 and Table S12). Interestingly, NTRs accounted for the majority of these genes: 91% in females and 71% in males.

Extensive evidence for sex-specific splicing was also observed in our data from genes expressed in both sexes. We used cufflinks-derived gene models (AAEL-NIPS and NTRs), which represent a significantly more complex transcriptome than the original AAEL gene set, to identify 2468 exons, originating from 1278 loci, as sex-differentially (exon is expressed significantly more abundantly in one sex *vs.* the other) or sex-specifically (exon is exclusively expressed in one sex) expressed with a false discovery rate of 0.05 (Table S13 and Table S14). Of the genes expressed sex-specifically, or those with exons expressed sex-specifically, a modest number (22 in males and 24 in females) are expressed beginning ~4−8 hrs into embryogenesis, suggesting that sex determination/sexual differentiation may have begun by this time. Male-specific genes with similar expression characteristics (although with no homology to those in Aedes) have also recently been identified on the Anopheles stephensi and Anopheles gambiae Y chromosomes, which are thought to be involved in sex determination in these insects (Hall *et al.* 2013).

One facet of gene regulation of particular interest because of its potential use in novel vector control strategies has to do with the mechanisms underlying sex-specific expression and mosquito sex determination. *A. aegypti* and other culicine mosquitoes lack heteromorphic sex chromosomes, with sex being controlled by an autosomal locus in which the male-determining allele, M, is dominant (Craig and Hickey 1967). The mechanism by which the M locus works to determine sex is unknown, but its activity presumably ultimately leads to sex-specific splicing of products of the doublesex locus, which is sex-specifically spliced in *Aedes* (Salvemini *et al.* 2011), and which controls somatic and gonad sexual development in many other insects (Gempe and Beye 2011). In a number of insects sex-specific splicing of dsx is regulated by sex-specific splice forms of tra (Gempe and Beye 2011). Interestingly, putative tra orthologs have not been identified in mosquitoes, and were not identified in our analysis, suggesting that regulation of sex-specific splicing of dsx may occur through a novel mechanism. Genes involved in sex determination may be included in the set of genes noted above with sex-specific expression or containing sex-specific exons (Table S13). However, it is also possible that some relevant genes are not yet included in the transcriptome, either because of low expression level, or because they are located in regions of the genome not yet included in the Aedes genome sequence, and thus not included in our transcriptome.

### TE dynamics

**Figure 3:**
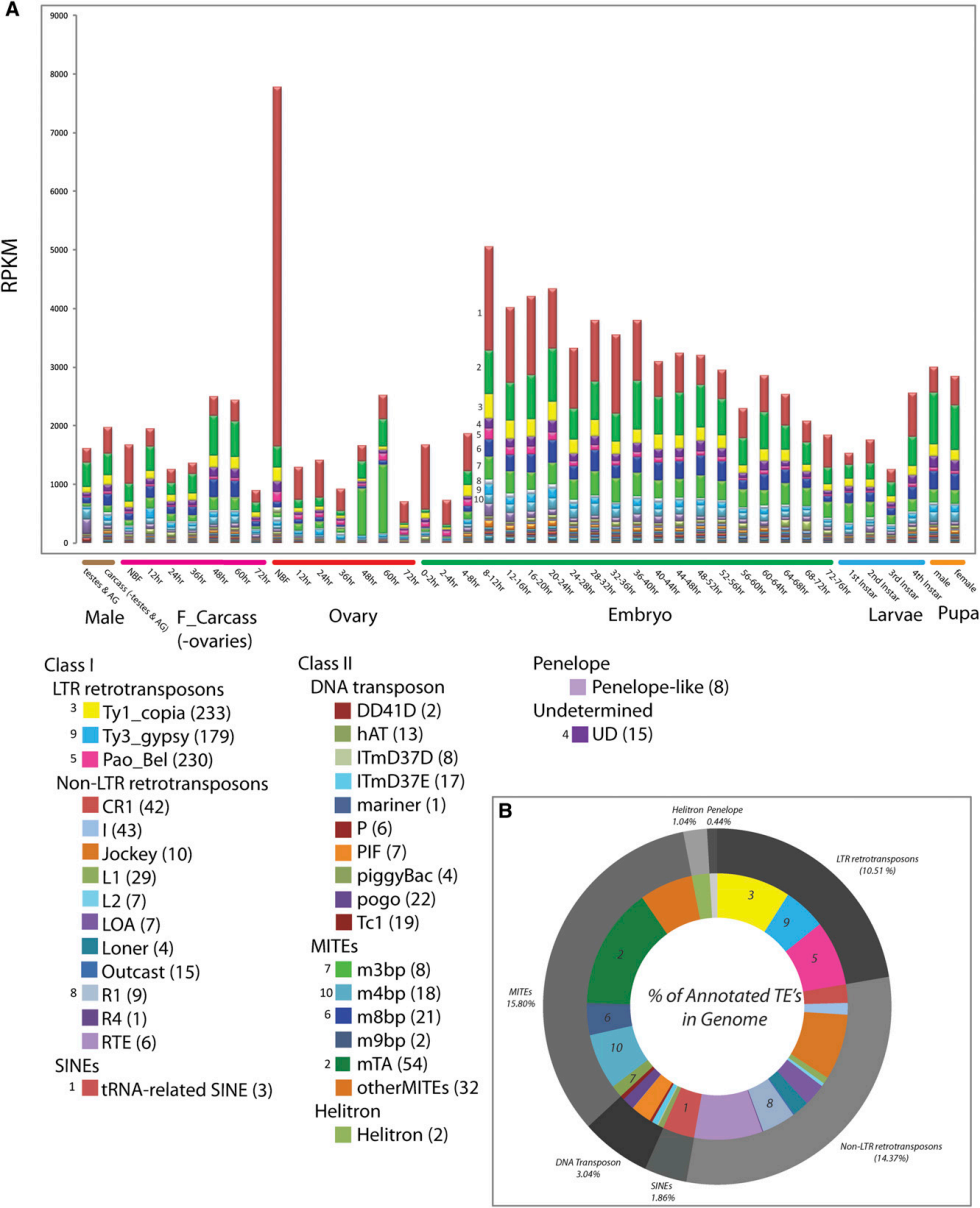
Developmental time course of TE expression profiles. Developmental expression profiles of different TE families, indicating RPKM values (y-axis) across all 42 developmental time points (x-axis). The top 10 TE families with the greatest expression levels are indicated from 1 (highest) to 10 (lowest). All families of TEs are indicated in the table in the lower left, with the number of elements in each family indicated in parentheses. Small numbers associated with specific boxes identify highly expressed TE families from upper plot of RPKM values (A). A circular pie chart indicating the percentage of the annotated genome occupied by each TE class (outer circle), and sublcass (inner circle) (B).

Almost 50% of the *A. aegypti* genome consists of identifiable TEs (Nene *et al.* 2007). TEs exist in a dynamic relationship with their host. They spread through mobilization in the host germline and must evade multiple host mechanisms designed to limit their amplification, which can decrease host fitness if left unchecked. To begin to understand the dynamics of TEs in *A. aegypti* we calculated the expression levels of transcripts derived from TEs through development (Figure 3, Table S15, and Table S16). The TE family with the greatest overall expression level is the tRNA-related SINE, members of which are expressed at especially high levels in the NBF ovaries. This family consists of three elements, including Feilai A, Feilai B (the most abundant TE in *A. aegypti* with ~50,000 copies per haploid genome), and gecko. There is a dramatic decrease in the expression level of all TEs, with the exception of the tRNA-related SINEs, in the late ovary (72-hr BF) and early embryo. This is followed by a pulse in expression of many TE families in the 4−8 hr and 8−12 hr embryo time points, followed by a progressive decrease through the completion of embryogenesis, and then an increase in expression in the late larvae and pupae, which may reflect expression in the developing germline.

### Complexity of small RNAs

Three major classes of small regulatory RNAs are microRNAs (miRNAs), endogenous small interfering RNAs, and Piwi-interacting RNAs (piRNAs) (Liu and Paroo 2010). miRNAs are initially transcribed as long RNAs containing hairpins. These can be processed through several different mechanisms, after which the released hairpin is transported to the cytoplasm, where it is cleaved by Dicer into a single short ~22 nt double-stranded RNAs. One or both of these strands are loaded into Argonaute family protein-containing complexes known as the RNA-induced silencing complex, where they act as guides for silencing of partially or completely complementary transcripts through translational repression and/or transcript degradation (Yang and Lai 2011). In contrast, siRNAs are derived from long double-stranded RNAs that are processed by Dicer to produce multiple small RNAs that are also loaded into Argonaute-containing RNA-induced silencing complex complexes (Okamura and Lai 2008). miRNAs range in length from 21 to 23 nt, whereas most siRNAs are ~21 nt. Finally, piRNAs are ~25−32 nt; their production does not require Dicer and is otherwise not well understood. They are defined by their association with members of the Piwi family of Argonaute proteins. piRNAs are generated from a variety of different precursors, including long single-stranded RNAs, and complex and repetitive regions, often carrying many transposons or transposon fragments (Siomi *et al.* 2011).

**Figure 4:**
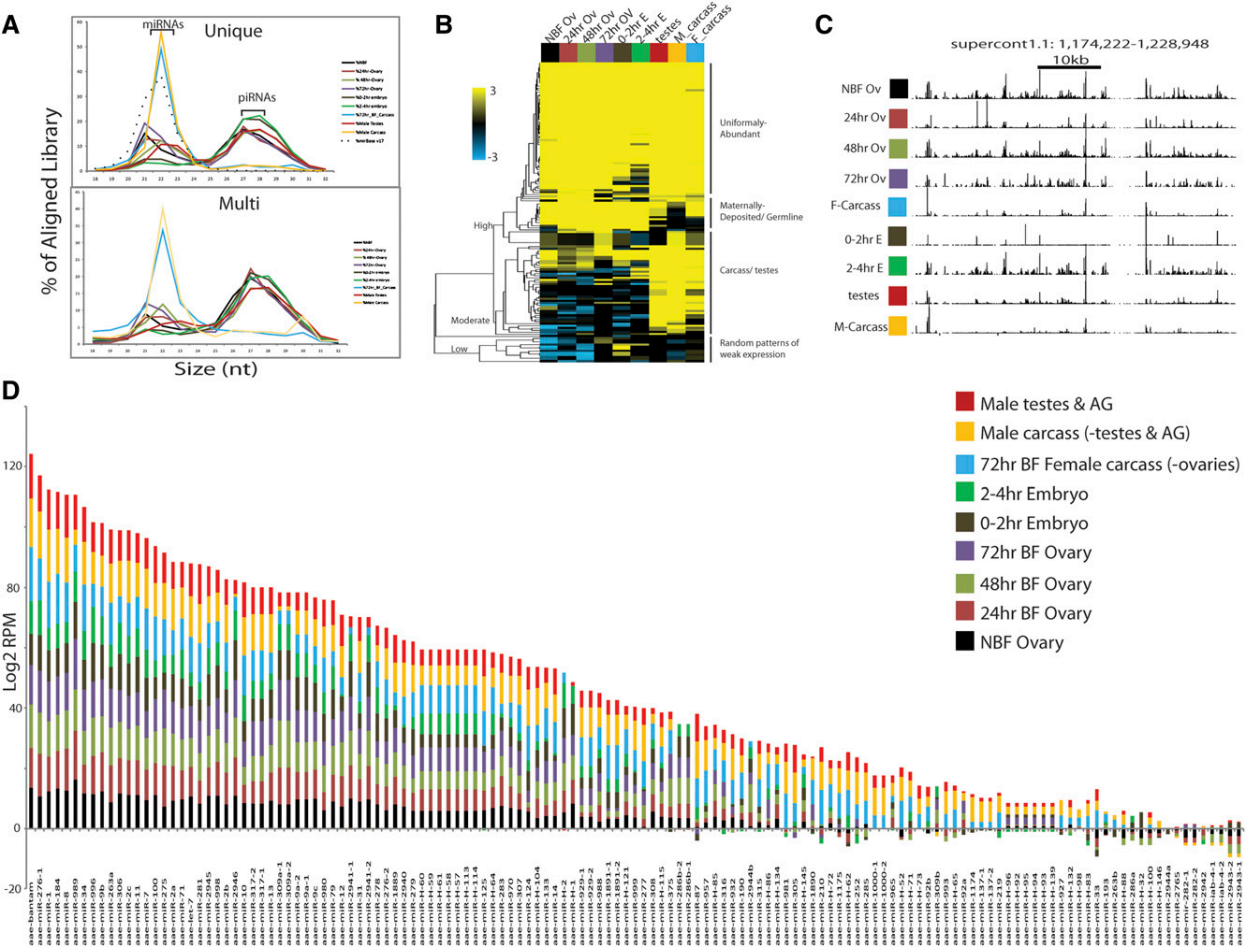
Small RNA distribution and clustering. Length distributions for small RNAs that map to the genome are indicated as percentages of the total reads mapping to the genome, for each library. Results from both unique and multimapping are shown. Samples are indicated to the right. Dotted line corresponds to the length distribution for previously annotated Aedes miRNAs included in mirBase (A). Heat map representation of all previously annotated miRNAs in *A. aegypti*, and 38 newly discovered miRNAs. Scale bar indicates FPKM Z-scores (B). Genome browser snap shot of a 54,727-bp genomic region, dense in 27−32bp mapped fragments, on supercontig 1: 1,174,222-1,228,948. All small RNA libraries are uniquely mapped (C). Color bar graph depicting the log_2_ RPM (reads per million) of each miRNA expressed in the 9 samples indicated to the right, organized in order from most to least abundant (D). The sample color scale for (A−C) is identical, as depicted in C, and is as follows: male testes and AG (red), male carcass (orange), 72-hr BF-F-carcass (blue), 2−4 hr embryo (green), 0−2 hr embryo (brown), 72-hr BF-ovary (purple), 48-hr Bf-ovary (light green), 24-hr BF-ovary (light red), and NBF ovary (black).

Depending on the sample, between 80 and 90% of the total small RNA reads produced mapped to the genome, either to unique sites or multiple positions (Table S3). Expression levels were merged into a master table in which 55,303,820 reads, 64.49% of the total mapped small RNA reads, formed 291,735 clusters covering 12MB of genomic sequence. Overall, 28,534,187 reads (51.6% of the total mapped reads) aligned to 37,712 genomic positions with a cluster length of 18−24 nt; 8,087,495 reads (14.6% of the total mapped reads) aligned to 151,806 genomic positions with a cluster length of 25−34 nt; and 18,682,168 reads (33.8%) aligned to 102,217 genomic positions with a cluster length >34 nt (Figure 4A and Table S17). The vast majority of clusters identified are expressed only (RPM = 0 in other tissues) in the ovary and early embryo samples (249,630; 85.56%). A much smaller number of clusters were specific to the 72-hr female carcass (2581; 0.88% of total clusters), the adult male carcass (415; 0.14% of total clusters) or the testes plus AG (13,227; 4.5% of total clusters).

**Figure 5:**
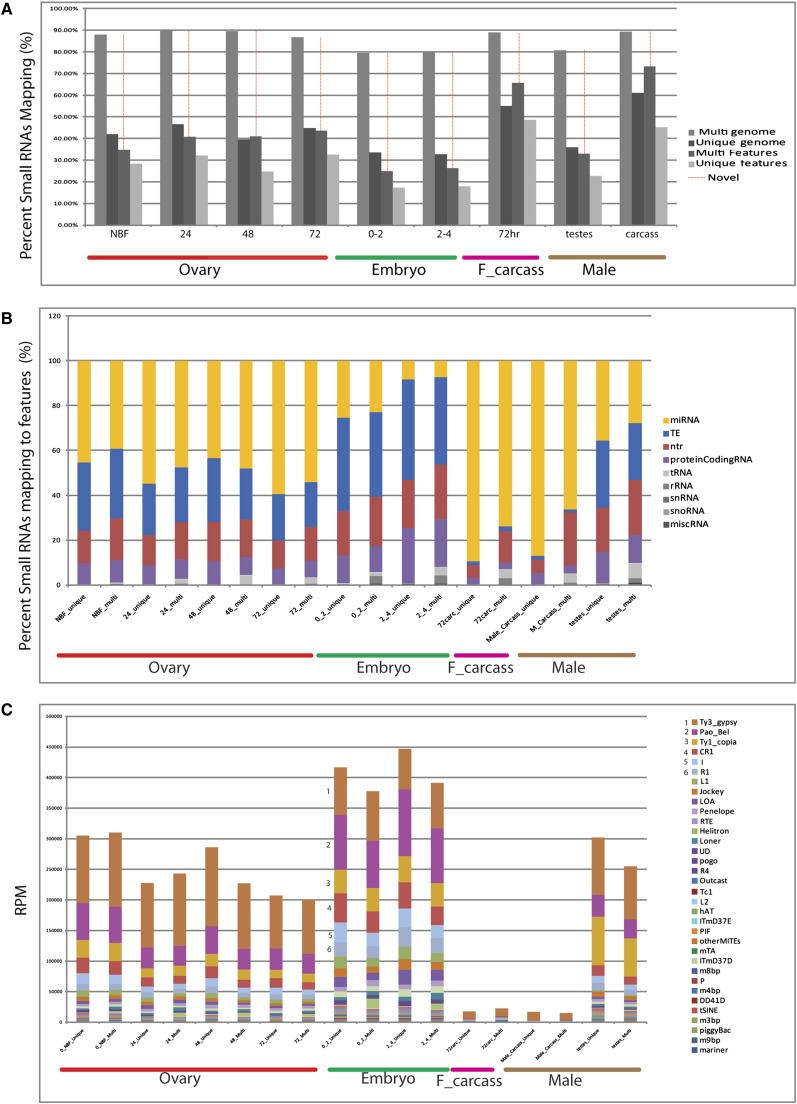
Small RNA mapping results and expression profiles to features. Percentage of reads mapping to the genome or annotated features; results are shown using multi-mapping (unlimited alignments) and unique mapping (single alignment) for the samples indicated on the X axis. The fraction of reads corresponding to novel transcribed features is indicated in red (A). Of the small RNAs that map to known features, the percent of small RNAs mapping to specific features is indicated for both multi and unique mapping (B). RPM values (y-axis) for each small RNA library (x-axis) were quantified against all annotated TE element families in *A. aegypti* for both multi and unique mapping. Numbers highlight the TEs for which small RNAs are most abundant across all samples (C).

Interestingly, depending on the sample, between 26.74 and 75.19% of the total clustered mapped reads, corresponding to 23.88 and 59.85% of the total reads respectively, mapped to regions of the genome with no other annotated features (Figure 5A). For those clusters that did map to annotated features, miRNAs, small RNA corresponding to sense or antisense fragments of transposons, polyAplus NTRs, and protein-coding mRNAs were the most abundant classes, with tRNAs, rRNAs, snRNAs, snoRNAs and miscRNAs making up the remainder (Figure 5B).

### Small RNA distributions

Small RNA libraries from different samples also have distinct size distributions. Male and female carcasses have a narrow distribution of lengths centered at 22 nt (Figure 4). As expected, most of these (~90%) are miRNAs (Figure 5B). In contrast, miRNAs comprise a much smaller fraction of the reads that map to known features in the germline and early embryo libraries, with many reads mapping to TEs, NTRs, and AAEL genes (Figure 5B). This shift in features to which the small RNAs map is particularly striking for libraries from 0−2 and 2−4 hr embryos, from which miRNAs comprise less than 25% or 10% of uniquely mapped reads, respectively. In addition to a peak of small RNA abundance centered around 22 nt, these libraries show a second broader peak RNA length centered around 27−28 nt. Most small RNAs mapping to TEs, which are known to be important sources of piRNAs in other organisms, fall within the size range 25−30 nt, suggesting that these are piRNAs. The RPM values for small RNAs mapping to specific TE element classes are shown in Figure 5C (Table S20). The general trend of small RNA abundance associated with specific TE families is consistent across the time points assayed, with the most abundant class mapping to Ty3-Gypsy, followed by Pao_Bel, Ty1_copia, CR1 and I. Very few small RNAs mapping to TEs are found in male and female carcasses, despite the fact that transposons are expressed in these tissues at significant levels (Figure 3A).

### miRNA identification

To identify miRNAs we aligned sequenced small RNAs to the most current miRBase database v18 using BLAST and identified 101 (100%) of previously annotated *A. aegypti* or *Aedes albopictus* miRNAs (Li *et al.* 2009; Skalsky *et al.* 2010). We also identified 36 novel *A. aegypti*−specific miRNAs, as well as two evolutionarily conserved miRNAs not previously identified in *A. aegypti* or *Aedes albopictus* (Table S18 and Table S19). Overall, 18% of the total small RNA reads mapping to the genome (25,241,117) aligned to previously annotated and newly predicted miRNAs, with an average mature miRNA size of 22 bp. The 15 most abundant miRNAs, each contributing >1.6% of total mapped reads, accounted for 81% of miRNA reads (Figure 4D). 95% of miRNAs were expressed in the ovaries, and 21% of the miRNAs are located in introns of annotated genes.

We compared the abundance of the miRNAs across all nine developmental time points and hierarchically clustered the data in a heat map to visualize prominent patterns of expression (Figure 5B, Table S18, and Table S19). The clustering produced three major groups of miRNAs: those with low, moderate and high expression levels. As subclasses within these groups we find (1) developmentally uniformly abundant, (2) carcass and testes biased, (3) ovary expressed and maternally deposited, (4) carcass and testes specific, and (5) various other patterns of expression, generally weaker. To visualize miRNA abundance, we plotted the normalized RPKM values for each miRNA across all nine developmental time points in a bar graph, ordered from most to least abundant. Bantam was the most abundant miRNA overall and in the adult carcasses, mir-996 was the most abundant miRNA in embryos, and mir-989 was the most abundant miRNA in the ovaries. mir-263B was expressed almost exclusively in the male and female carcasses, whereas mir-286b-1, mir-286-b-2, mir-309b, mir-2944b, and mir-286a were specifically expressed in the female ovary and maternally deposited into the early embryos (Figure 5D).

### piRNAs

In *Drosophila*, a number of piRNAs derive from clusters composed of repeated sequences and transposon remnants that are localized in pericentromeric and subtelomeric regions (Brennecke *et al.* 2007; Saito *et al.* 2006). To search for dense regions of piRNA expression, we grouped together all regions expressing small RNAs within 1 kb of each other, with a minimum read count of three, into single superclusters. This reduced the total cluster number by 85%, producing a total of 46,631 superclusters ranging from 44 to 54,727 bp (avg. 992 bp) covering 43 MB of genomic sequence (Table S21). The majority of reads in these large clusters are between 24-32 nt in length, suggesting that these represent piRNA clusters. An example of a 54,727-bp region to which no known miRNAs or annotated genes align, that is dense with uniquely mapped 24-32bp small RNAs in all assayed tissues is illustrated in Figure 5C. A recent analysis of small RNAs from whole *A. aegypti* adults also resulted in the identification of large genomic regions that express many 24-32 nt small RNAs (Arensburger *et al.* 2011). Our results extend these observations through analysis of both germline and somatic tissue samples.

**Figure 6:**
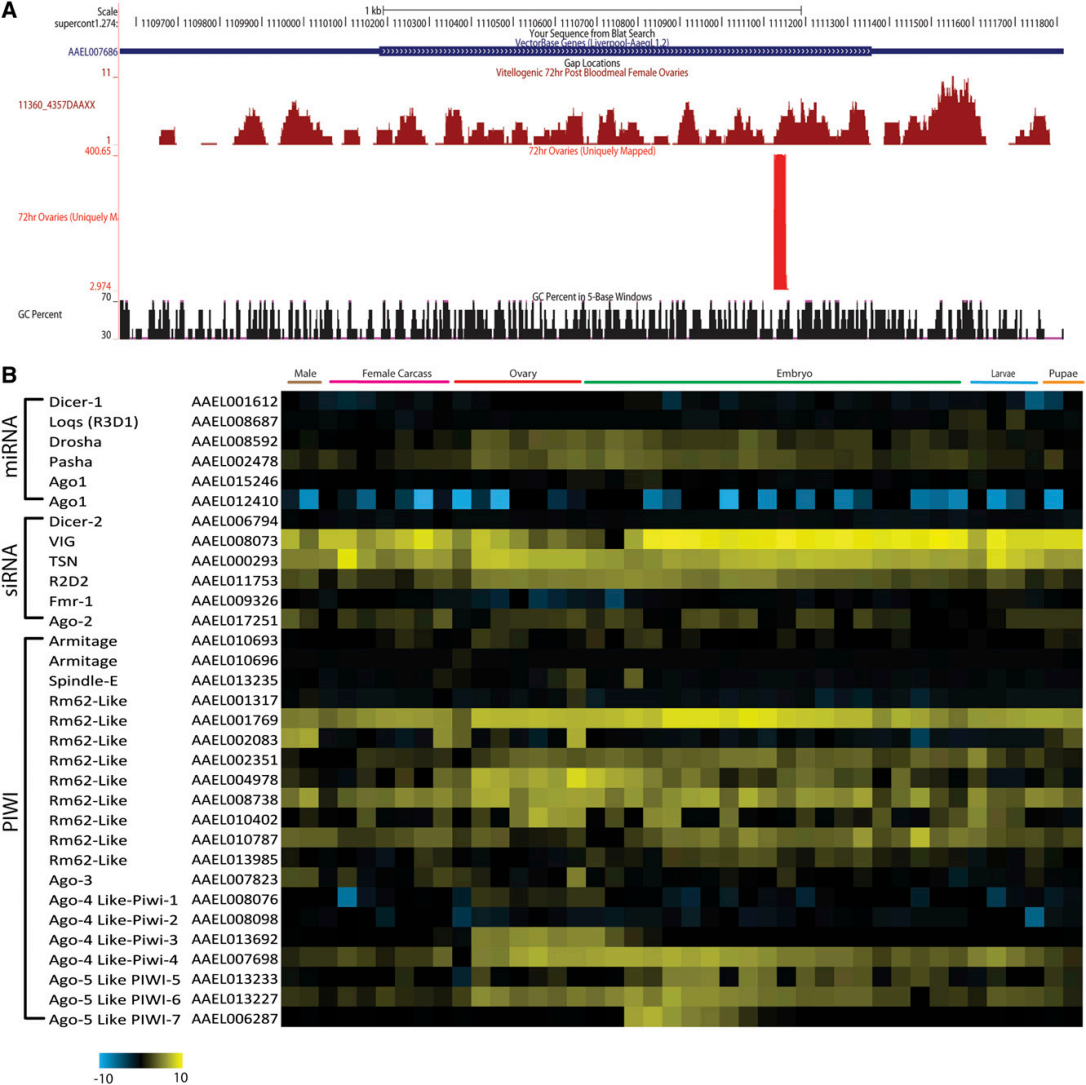
piRNA production by a single locus and RNA expression profiles for genes involved in small RNA production. (A) An example of piRNAs mapping specifically to the 3′UTR of AAEL007686 is shown. (B) The expression dynamics across development of genes important for processing of different small RNAs, including miRNA, siRNAs, and piRNAs are shown as a heat map (log2 FPKM).

Other piRNA clusters derive from unique positions within genes. For example, in *Drosophila*, traffic jam (tj) encodes a large Maf factor, which is expressed in the ovarian soma and required for gonad morphogenesis. Numerous piRNAs are produced from the sense strand of the tj 3′UTR (Robine *et al.* 2009; Saito *et al.* 2009). Small RNAs of a similar length are also produced from the 3′UTR of the *A.aegypti* tj homolog (AAEL007686), and from 7736 AAEL mRNAs with at least three small RNA reads mapping in at least one tissue (Figure 6A and Table S22), suggesting that piRNAs regulation of protein coding genes in Aedes is widespread. Similar results were reported by Arensburger *et al.* (2011).

To gain a global view of the genes involved in small RNA processing we constructed a heat-map to visualize their expression dynamics across development (Figure 6B). Increased expression of a number of genes occurs in the ovary in response to a blood meal. Other patterns of tissue/stage specific expression are also apparent. For example, Piwi-3 is specific to the ovary, whereas Piwi-7 is zygotically activated specifically in the early embryo. The significance of these patterns is unknown, but they suggest that small RNA processing is dynamically regulated, and that much remains to be learned about small RNA processing and function.

### Transcriptional profiling and transgenesis-based vector control

In addition to providing a tool for basic research on *A. aegypti*, the developmental transcriptome will facilitate the development of transgenesis-based control of vector populations through population suppression or replacement of the wild population with individuals refractory to disease transmission. For example, one class of suppression strategies involves the creation of males whose sperm express a transgene-based toxin that cause the death of all progeny (Klein *et al.* 2012; Windbichler *et al.* 2008). In a second, males are engineered to carry a transgene expressed (in a repressible manner in the laboratory) in females, causing their death or some other large fitness cost such as flightlessness (Fu *et al.* 2010). Promoters from genes that drive testis- or female-specific expression are needed for these approaches, and several have been identified in *Aedes aegypti* and *Anopheles gambiae* using candidate gene approaches (reviewed in Catteruccia *et al.* 2009). Table S11 (female specific) and Table S12 (male specific) identify a number of additional genes with similar expression patterns that may also prove useful. Exons of genes expressed in both sexes that have strict sex-specific splicing of particular exons can also be used to bring about sex-specific phenotypes (Fu *et al.* 2010). Examples of these are found in Table S13. A third population suppression strategy involves driving a engineered homing endonuclease into a population, with the insertion site for the homing endonuclease being located within gene whose product is required for female viability or fertility (Burt 2003; Windbichler *et al.* 2011). Homing endonucleases have very long target sites and are highly specific in their insertion site preference. Although number of novel homing endonucleases have recently been identified (Takeuchi *et al.* 2011), and some progress has been made in creating homing endonucleases with altered target specificity (Ashworth *et al.* 2010; Ulge *et al.* 2011), many genes lack sequences that would make them targets for cleavage by currently available homing endonucleases. In addition, cleavage followed by nonhomologous end joining (in the absence of homing) can potentially result in destruction of the target site without loss of gene function (Burt 2003; Deredec *et al.* 2008). Therefore, it is important to have many potential target genes available. Good candidates are likely to be found among those genes expressed only in the female (Table S24 and Table S11). Modeling studies suggest that ideal targets would be genes whose loss-of-function results in recessive, but not dominant fitness costs, in somatic tissues, but not the germline (Deredec *et al.* 2008). For such genes, homing in the germline of a heterozygous female (which creates homozygous mutant germ cells) does not compromise transmission of the HEG to the next generation (Deredec *et al.* 2008). Genes expressed specifically in female somatic tissues are indicated in Table S24.

Several strategies have also been proposed for driving genes for disease refractoriness into wild mosquito populations, including the use of engineered homing endonucleases (Burt 2003), engineered zygotic underdominance (Davis *et al.* 2001), male meiotic drive (Sinkins and Gould 2006), *Medea* (Chen *et al.* 2007), and a Medea-related gene drive system known as UD^MEL^ (Akbari *et al.* 2013). Here we focus on *Medea* and UD^MEL^ as these have been successfully implemented in *Drosophila* (Akbari *et al.* 2013; Chen *et al.* 2007). A *Medea* selfish genetic element consists of two linked genes: a toxin that is expressed only in the female germline, with effects that are passed to all progeny, and a neutralizing antidote, expressed under the control of an early zygote-specific promoter. As implemented in *Drosophila*, the toxin consists of maternally-expressed miRNAs that silence the expression of a maternally expressed gene whose product is required for embryogenesis, thereby creating a maternal-effect lethal phenotype in all progeny. The antidote is a zygotically expressed version of the maternally silenced gene, resistant to miRNA-dependent silencing, able to provide the missing maternal product, thereby restoring normal development (Akbari *et al.* 2013; Chen *et al.* 2007). *Medea* spreads because when present in females, it causes the death of all progeny that fail to inherit it, thereby promoting its spread at the expense of homologous chromosomes that lack it (Wade and Beeman 1994; Ward *et al.* 2011). In the UD^MEL^ system, which has a higher introduction threshold, and is therefore more easily recalled from the population, similar components are used, but they are located on different chromosomes. miRNAs expressed and processed at high levels in the ovary and early embryo are good candidates to function as backbones for toxin expression in both systems (Table S18 and Table S19).

Transcripts found in the ovary and early embryo are likely to derive from genes that are expressed in the maternal germline, with their products being dumped into the oocyte and persisting for some time following fertilization. To identify genes expressed specifically in the female germline whose promoters could be used to drive the expression of toxins, we filtered the developmental transcriptome to identify genes expressed only in the ovary and early embryo (RPKM = 0 in other samples), with the embryonic expression trending only downward following fertilization. Many AAEL genes and NTRs with these expression characteristics were identified (Table S25). Transcripts found only in the ovary and not in the early embryo are likely to derive from genes expressed in ovarian somatic tissues such as the follicle cells, which die before egg laying. Filtering of the transcriptome to identify genes strictly expressed in the ovary but not elsewhere (RPKM = 0 in female and male carcass, testes plus AG and 0−2 hr embryo) identified several genes with this expression characteristic (Table S26). These genes and their promoters would not be useful for generation of Medea elements, but their transcription units could be interesting targets for homing endonucleases designed to impair female fertility.

Genes that are expressed ubiquitously, specifically in the early zygote, can provide promoters to drive the expression of antidotes. In *Aedes aegypti* zygotic transcription is first seen ~2−4 hrs post oviposition, and two kinesin light chain genes, AaKLC2.1 and AaKLC2.2, have been shown to be expressed specifically from 2 to 8 hrs, although whether these genes are expressed ubiquitously in space is unknown (Biedler and Tu 2010). Several other genes with early zygotic expression in Aedes have also been recently identified, though the results of our sequencing would suggest that some of these are also expressed at significant levels in stages not sampled (Biedler *et al.* 2012). To provide a comprehensive list of genes expressed only in the early zygote we filtered the *Aedes aegypti* developmental transcriptome to identify genes that were not expressed (FPKM = 0) in the 7 ovarian samples or 0−2hr embryos, that were expressed in the 2-4hr time window, and that then decreased in intensity, as expected for transcripts expressed transiently and then degraded. This analysis discovered 40 strictly early zygotic AAEL genes and NTRs, validating the expression patterns of the kinesin light chain genes and identifying 38 additional genes and NTRs (Table S27).

## Discussion

In this study we used RNA-seq to provide the most comprehensive description to date of the transcriptome of a mosquito vector of disease, *A. aegypti*. Our analysis of polyA plus libraries revealed an unanticipated complexity to the *Aedes* transcriptome. Our results provide confirmation for 99% (15,941/15,988) of previously annotated AAEL genes. However, they also provide evidence for extensive transcription in other areas of the genome. Approximately11.19 MB of this is associated with genomic sequence overlapping the AAEL genes (AAEL-NIPs). Some of this may reflect the identification of new genes inside or overlapping with AAEL genes, while other new sequence may reflect the identification of novel transcribed AAEL exons. NTRs that show no overlap with AAEL genes or AAEL-NIPs provide expression evidence for another 13.27 MB of transcribed DNA. The validity of these new predictions will need to be confirmed using other techniques, but the fact that many of these novel transcripts and NTRs were observed in multiple samples suggests that many new splice forms of previously annotated genes and new genes have in fact been identified. In addition, our analysis is almost certainly an underestimate of the fraction of the genome transcribed as long RNA, since rare transcripts found only in specific tissues may not have been identified. Transcripts will also have been missed if they are only expressed in specific contexts—such as during stress, aging, exposure to toxins or pathogens—that were not sampled, or if they are not polyadenylated.

Roughly 21.5% of the NTRs were identified as being potentially noncoding, based on having a size greater than 200 nt, an FPKM of 10 or greater, and the lack of an ORF of 200 amino acids or greater. This set of cutoffs is somewhat arbitrary as it will incorrectly call as noncoding transcripts encoding small peptides or proteins generated through stop codon readthrough (Jungreis *et al.* 2011; Ladoukakis *et al.* 2011), and it will exclude short noncoding NTRs. However, it has been used as a part of search strategies for high confidence noncoding NTRs in other systems (Young *et al.* 2012). The structures of these NTRs are quite distinct in that their transcript lengths are on average shorter than AAEL genes and AAEL-NIPs (NTRs = 1132 bp; AAEL = 1514 bp; AAEL-NIPs = 4051 bp), and have fewer exons (NTRs = 1.58; AAEL = 3.79; AAEL-NIPs = 7.65) in addition to their genomic length being shorter (NTRs = 9250 bp; AAEL = 14,888 bp; AAEL-NIPs = 72,828 bp). Some NTRs may simply represent exons of coding genes that have not been linked to AAEL or AAEL-NIPs because of sequencing depth. However, a number are expressed at high levels, making this explanation unlikely as a general conclusion. It is also possible that some represent contamination by genomic DNA. Although it is difficult to exclude this possibility in the case of single exon transcripts, we note that a number of these NTRs are expressed differentially, during the lifecycle, making this also unlikely as a general explanation (Figure S1). A more rigorous identification of noncoding RNAs among the *Aedes aegypti* NTR set will be facilitated by use of comparative genomics methods (Lin *et al.* 2011; Washietl *et al.* 2011), once RNAseq-based transcriptomes are available for closely related species such as *A. albopictus*, and more distantly related species such as *Anopheles gambiae* and *Anpheles stephensi*.

Our cluster analysis and characterization of sex-differential and sex-specific expression of AAEL genes, AAEL-NIPs and NTRs identified a number of patterns of co-regulated gene expression. Analysis of these patterns will help to uncover *cis*-acting regulatory elements, the genetic circuits underlying specific developmental transitions, biology specific to adult males and females, and parts needed to engineer transgene-based mechanisms capable of bringing about population suppression or replacement. The patterns observed are likely to often represent changes in the transcription and splicing of the genes involved. However, differential regulation of transcript stability by *trans*-acting factors such as microRNAs and RNA-binding proteins will also undoubtedly be important in some cases. In addition, recent work has shown that adults of Aedes strains differing in a number of ways, including place of origin, number of generations in the lab, and susceptibility to dengue infection, show significant differences in their transcriptional responses to sugar feeding or a blood meal (Bonizzoni *et al.* 2012b). Thus, the observations presented here should be taken to constitute only a broad-brush overview of the *A. aegypti* developmental transcriptome, albeit of a strain (Liverpool) used as a model by many researchers. An important future goal will be to characterize patterns of gene expression from many strains, with the goal of identifying developmental and adult signatures that predict important life history traits such as longevity, ecotype, resistance to agents that might be used in larval or adult vector control, and susceptibility to dengue infection. The pathways identified by these genes may provide new targets for transgenesis-based strategies of vector control and/or vector competence.

One of the most remarkable features of the Aedes aegypti genome is its large TE content (~50% of the genome), and overall size, 1.37 Gigabases, almost five times that of the malaria vector Anopheles gambiae. Our analysis highlights the dynamic nature of TE expression throughout the Aedes lifecycle. Changes in the levels and relative abundance of specific TEs during adult ovarian development in response to blood feeding are particularly striking. Because the female germline is a target for transposon spread, it will be particularly interesting to determine if TEs with abundant transcripts are undergoing active expansion in Aedes. Related to this last point it is also noteworthy that some of the TEs with the highest levels of expression (tSINE, mTA, m8bp, and m3bp) (Figure 3A) have very low numbers of corresponding small RNAs (Figure 4C). This is particularly obvious for the tRNA-related SINEs. These are expressed at very high levels in the nonblood-fed ovary, and are abundantly expressed in other samples as well. Yet small RNAs corresponding to these elements make up only between 0 and 0.004% of the total TE-associated small RNAs. MITES also have low levels of small RNAs, despite having high expression levels, and high copy numbers in the genome. Low levels of piRNAs targeting these TE classes were also noted by Arensburger *et al.* (Arensburger *et al.* 2011). These authors suggested lack of coding potential as one possible explanation for such a pattern and noted relatively more siRNAs targeting these elements. Although we also see a modest bias toward siRNAs the fact remains that these elements are simply not targeted at high frequency, or do not act as sources of small RNAs, in all samples tested. One possibility is that these invaded only recently, and have not yet been captured by piRNA-generating loci. More detailed studies of the evolutionary history of these elements will be required to test this hypothesis, but initial characterization of the Feilai family of tRNA-related SINE elements shows significant sequence diversity, suggesting they are unlikely to be of very recent origin (Tu 1999). Overall, our analysis highlights the dynamic nature of TE expression throughout development.

Our results show that the Aedes small RNA transcriptome is dynamically regulated. Depending on the sample, we observed that between 24.81 and 65.58% of small RNA reads mapped to annotated features. Of those that map to features there are dramatic shifts in the nature of the features targeted depending on the sample. miRNAs predominate in somatic carcass tissues, with NTRs making up the next highest category. In the ovary and embryo samples, 71.01–82.19% of reads that do not map to features are of size range 26−32 bp, suggesting they are piRNAs. Similar observations were made by Arensburger *et al.*, in samples from whole adults (Arensburger *et al.* 2011); our work refines these observations to multiple tissues, both germline and somatic. Some piRNAs that do not map to features may be degenerate remnants of past battles with extinct transposons. Alternatively, and/or in addition, some may arise simply because specific regions of the genome are predisposed to generate piRNAs, with whatever is inserted into the locus becoming a substrate for piRNA production. In such a model, these regions would act to sample and respond to TEs (and other sequences) that happen to insert into them in a manner similar to the Crispr loci in prokaryotes, which generate small RNAs for genome defense from sequences that become inserted into them (Barrangou 2013). It will be particularly interesting to compare the Aedes piRNA system with that of anopheles mosquitoes to see if this sheds light on the mechanisms leading to the massive expansion of the Aedes TE content and genome size.

We have incorporated the data from our analysis into a web-based genome browser structured using the UCSC browser format (Kent *et al.* 2002). We include the current AAEL annotations as well as new AAEL-NIPs, NTRs and transcript models predicted using Cufflinks and Tophat. Each transcript and gene model is linked to Vectorbase, which provides reference genome sequences and other information on the most important disease vectors (Lawson *et al.* 2009). Researchers can view *Aedes aegypti* gene expression data for every time point, explore transcript and gene models, and compare sequence conservation with several Drosophila species and Anopheles gambiae and *Culex quinquefasciatus*. Data can also be imported into the genome browser for viewing and comparison. As an example, we incorporated recent RNA-seq data describing patterns of gene expression observed 5 hr PBM in whole *Aedes aegypti*, a time point not included in our data set (Bonizzoni *et al.* 2011). This browser can be found at http://Aedes.caltech.edu. The transcriptome data presented will facilitate study of Aedes basic biology. It will also facilitate the development of transgenesis-based strategies of population suppression or population replacement.

## Supplementary Material

Supporting Information
