# Supplementary material for: The Developmental Transcriptome of the Mosquito Aedes aegypti, an Invasive Species and Major Arbovirus Vector
Source: G3 (Bethesda). 2013 Sep 1;3(9):1493–509. doi: 10.1534/g3.113.006742 (PMC3755910; doi:10.1534/g3.113.006742)
Supplement: Supporting Information [file supp_3_9_1493__index.html]

The Developmental Transcriptome of the Mosquito Aedes aegypti, an Invasive Species and Major Arbovirus Vector — Supporting Information 

# The Developmental Transcriptome of the Mosquito *Aedes aegypti*, an Invasive Species and Major Arbovirus Vector

## Supporting Information for Akbari *et al.*, 2013

**Files in this Data Supplement:**

- Supporting Information - Figures S1 and S2, Files S1-S4, and Tables S1-S30 (PDF, 697 KB)
- Figure S1 - Soft Clustering of NTRs (PDF, 1.4 MB)
- Figure S2 - Scatter plots showing sex-biased gene expression (PDF, 184 KB)
- File S1 - New Isoforms of Annotated genes GTF (.zip, 1.8 MB)
- File S2 - NTR GTF (.zip, 423 KB)
- File S3 - Aggregated AAEL loci (.zip, 668 KB)
- File S4 - Non-coding NTRs GTF (.zip, 93 KB)
- Table S1 - Summary of Sequenced Experimental Datasets (.xlsx, 13 KB)
- Table S2 - Poly(A+) multi-map read and mapping statistics (.xlsx, 18 KB)
- Table S3 - Aedes Small RNA multi-map read and mapping statistics (.xlsx, 10 KB)
- Table S4 - NTRs Blast and Interpro (.xlsx, 1.7 MB)
- Table S5 - NTR long ORF interpro domains (.xlsx, 13 KB)
- Table S6 - NTR non-coding fasta (.xlsx, 818 KB)
- Table S7 - NTR Fuzzy Membership (.xlsx, 89 KB)
- Table S8 - Complete Developmental Transcriptome Transcripts (.xlsx, 26.9 MB)
- Table S9 - Complete Developmental Transcriptome Genes (.xlsx, 12.4 MB)
- Table S10 - AAEL genes mFuzz cluster memebership (.xlsx, 804 KB)
- Table S11 - Female specific Genes and NTRs (.xlsx, 61 KB)
- Table S12 - Male specific Genes and NTRs (.xlsx, 14 KB)
- Table S13 - Sex differentially expressed exon parts (.xlsx, 320 KB)
- Table S14 - Interpro scan hits for differentially expressed exon parts (.xlsx, 52 KB)
- Table S15 - Transposable element Family expression (.xlsx, 33 KB)
- Table S16 - Transposable Elements Expression (polyA+) (.xlsx, 1.5 MB)
- Table S17 - Uniquely-Mapped Small RNA clusters (.xlsx, 50.4 KB)
- Table S18 - miRNA expression multi-map (.xlsx, 54 KB)
- Table S19 - miRNA expression unique-map (.xlsx, 47 KB)
- Table S20 - Transposable Elements Expression (smallRNA) (.xlsx, 164 KB)
- Table S21 - Aggregated Clusters 1kb (smallRNA) (.xlsx, 2.6 MB)
- Table S22 - Small RNAs mapped to Protein Coding genes (.xlsx, 4.1 MB)
- Table S23 - Small RNA cloning primers (.xlsx, 12 KB)
- Table S24 - Female Somatic Specific genes and NTRs (.xlsx, 11 KB)
- Table S25 - Strictly Maternal Genes and NTRs (.xlsx, 14 MB)
- Table S26 - Ovary specific Genes and NTRs (.xlsx, 145 KB)
- Table S27 - Early Zygotic Genes and NTRs (.xlsx, 20 KB)
- Table S28 - Female 20x upregulated Genes and NTRs (.xlsx, 110 KB)
- Table S29 - Male 20x upregulated Genes and NTRs (.xlsx, 14 KB)
- Table S30 - Gene Ontologies clusters 1-20 (.xlsx, 316 KB)
