# Supplementary material for: The Developmental Transcriptome of the Mosquito Aedes aegypti, an Invasive Species and Major Arbovirus Vector
Source: G3 (Bethesda). 2013 Sep 1;3(9):1493–509. doi: 10.1534/g3.113.006742 (PMC3755910; doi:10.1534/g3.113.006742)
Supplement: Supporting Information [file supp_g3.113.006742_006742SI.pdf]

G3·Genes | Genomes | Genetics

The Developmental Transcriptome of the Mosquito *Aedes aegypti*, an invasive species and major arbovirus vector.

Omar S. Akbari*, Igor Antoshechkin*, Henry Amrhein, Brian Williams, Race Diloreto, Jeremy Sandler and Bruce A. Hay.

Division of Biology, MC 156-29, California Institute of Technology, Pasadena, CA 91125, USA

Key words: *Aedes aegypti*; Dengue Fever; yellow fever; Chikungunya; Malaria; Population Replacement; Transcriptome; Medea; Gene Drive

*Equal Contribution

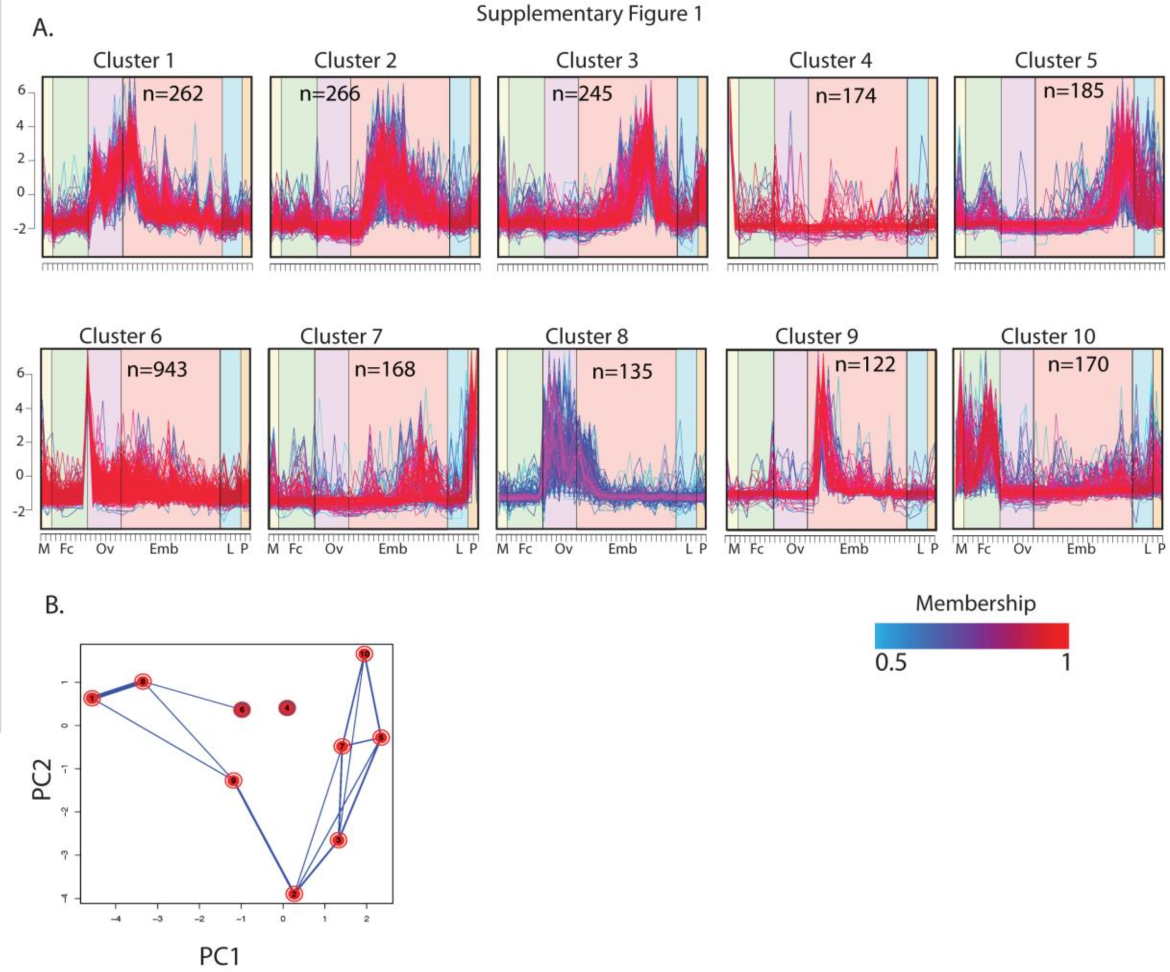

Figure S1 Soft Clustering of NTRs. 10 NTR expression profile clusters are identified through soft clustering procedure. Each NTR is assigned a line color corresponding to its membership value, with red (1) indicating high association. The major developmental groups are organized as in Figure 1B-D (A). Principal component analysis shows relationships between the 20 clusters, with thickness of the blue lines between any two clusters reflecting the fraction of genes that are shared (B, thickness of blue lines). n= the number of genes in each cluster.

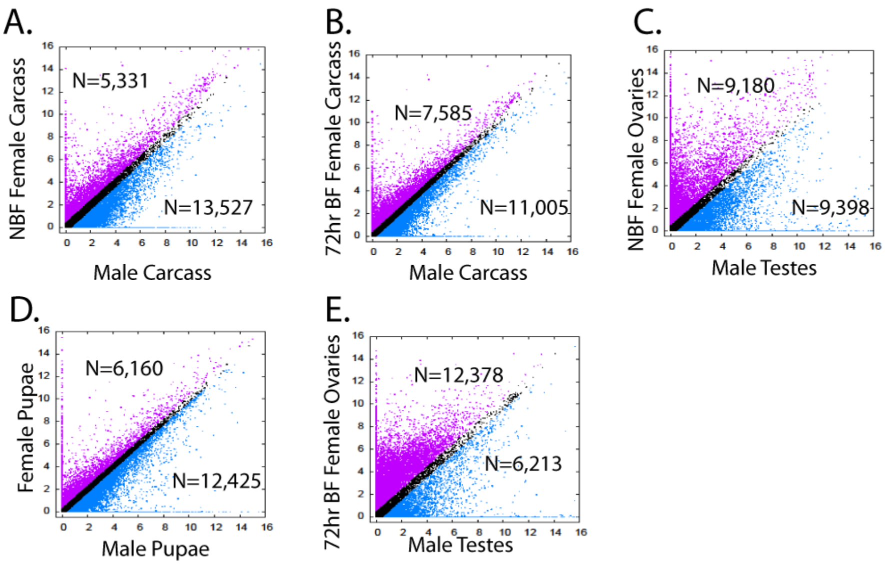

Figure S2 Scatter plots showing sex-biased gene expression. Scatter plots showing sex-bias in log(2) for AAEL genes and NTRs. 50% of the data is unbiased (black). Male biased gene expression is indicated in blue, with points falling on the X-axis showing male-specific expression. Female biased gene expression is indicated in purple, with points falling on the Y-axis showing female-specific expression. N indicates the number of genes in each category. The comparisons include non blood-fed female carcass vs. male carcass (A), female carcass 72hrs post blood feed vs. male carcass (B), non blood fed ovaries vs. testes & AG (C), female pupae vs. male pupae (D), and 72hr PBM ovaries vs. testes & AG (E). Lists of male and female sex specific genes can be located in the supplement (supplementary tables 24, 25).

Files S1-S4

Supporting Files

Available for download as compressed files at http://www.g3journal.org/lookup/suppl/doi:10.1534/g3.113.006742/-/DC1.

File S1. New Isoforms of Annotated genes GTF. A gene transfer format (GTF) file for the new isofroms of annotated genes.

File S2. NTR GTF.
A GTF file for all the NTRs.

File S3. Aggregated AAEL loci. A GTF file for the annotated AAEL genes which aggregate into loci.

File S4. Non-coding NTRs GTF. A GTF file for all the predicted non-coding NTRs.

Tables S1-S30

Supporting Tables

Available for download at http://www.g3journal.org/lookup/suppl/doi:10.1534/g3.113.006742/-/DC1.

Table S1. Summary of Sequenced Experimental Datasets. Summary of samples sequenced and type of sequencing preformed: short poly(A+), paired-end poly(A+), small-RNA.

Table S2. Poly(A+) multi-map read and mapping statistics. Mapping statistics including percentages for each poly (A+) sample indicating the total reads produced, total reads mapped to junctions, total reads mapped to exons, total reads mapped uniquely, total reads mapped to multiple locations, and total reads mapped.

Table S3. Aedes Small RNA multi-map read and mapping statistics. Mapping statistics for all the small RNA seq samples including total reads, total reads mapped, total reads mapped uniquely and multiply.

Table S4. NTRs Blast and Interpro. Blast and interpro results for all the novel NTRs indicating top blast hits with e values.

Table S5. NTR long ORF interpro domains. Interproscan results indicating the protein domains discovered in 14 NTRs.

Table S6. NTR non-coding fasta. Fasta file of 3070 predicted non-coding NTRs.

Table S7. NTR Fuzzy Membership. Fuzzy cluster membership values for each NTR in supplementary figure 1.

Table S8. Complete Developmental Transcriptome Transcripts. FPKM expression values for all known and discovered transcripts across all 42 developmental timepoints.

Table S9. Complete Developmental Transcriptome Genes. FPKM expression values for all known and discovered genes across all 42 developmental timepoints.

Table S10. AAEL genes mFuzz cluster memebership. Fuzzy cluster membership values for each annotated gene in figure 2.

Table S11. Female specific Genes and NTRs. A list of genes and NTRs with expression in the female samples and no detected expression in the male samples (FPKM=0).

Table S12. Male specific Genes and NTRs. A list of genes and NTRs with expression in the male samples and no detected expression in the female samples (FPKM=0).

Table S13. Sex differentially expressed exon parts. The exons that are differentially expressed are indicated in addition to the p-value, mean base and log2 fold ratio of expression between male and female and FDR of 0.05.

Table S14. Interpro scan hits for differentially expressed exon parts. Interpro scan hits with descriptions and associated gene ontologies for each differentially expressed exon.

Table S15. Transposable element Family expression. FPKM expression of each transposable element family across all 42 developmental time points.

Table S16. Transposable Elements Expression (polyA+). FPKM expression of each individual transposable element across all 42 developmental time points.

Table S17. Uniquely-Mapped Small RNA clusters. A list of 291,735 small RNA clusters identified with expression values and read counts, genomic coordinates and cluster size.

Table S18. miRNA expression multi-map. The expression levels for all miRNAs quantified by allowing reads to map multiply (unlimited) to the genome.

Table S19. miRNA expression unique-map. The expression levels for all miRNAs quantified by allowing reads to map uniquely (only once) to the genome.

Table S20. Transposable Elements Expression (smallRNA). Expression levels of small RNAs mapped to transposable elements.

Table S21. Aggregated Clusters 1kb (smallRNA). Small RNAs which are within 1kb distance from each other were aggregated together to form larger clusters.

Table S22. Small RNAs mapped to Protein Coding genes. Expression levels of small RNAs mapped to protein coding genes.

Table S23. Small RNA cloning primers. List of the primers used to clone the small RNA libraries.

Table S24. Female Somatic Specific genes and NTRs. A list of Genes and NTRs greater then or equal to 200bp with an average FPKM value of 10 or greater in the summed female somatic samples (female carcass') that were not expressed in male samples.

Table S25. Strictly Maternal Genes and NTRs

Table S26. Ovary specific Genes and NTRs

Table S27. Early Zygotic Genes and NTRs

Table S28. Female 20x upregulated Genes and NTRs

Table S29. Male 20x upregulated Genes and NTRs

Table S30. Gene Ontologies clusters 1-20.
