# Supplementary material for: The Developmental Transcriptome of the Mosquito Aedes aegypti, an Invasive Species and Major Arbovirus Vector
Source: G3 (Bethesda). 2013 Sep 1;3(9):1493–509. doi: 10.1534/g3.113.006742 (PMC3755910; doi:10.1534/g3.113.006742)
Supplement: Supporting Information [file supp_g3.113.006742_FigureS1.pdf]

Supplementary Figure 1

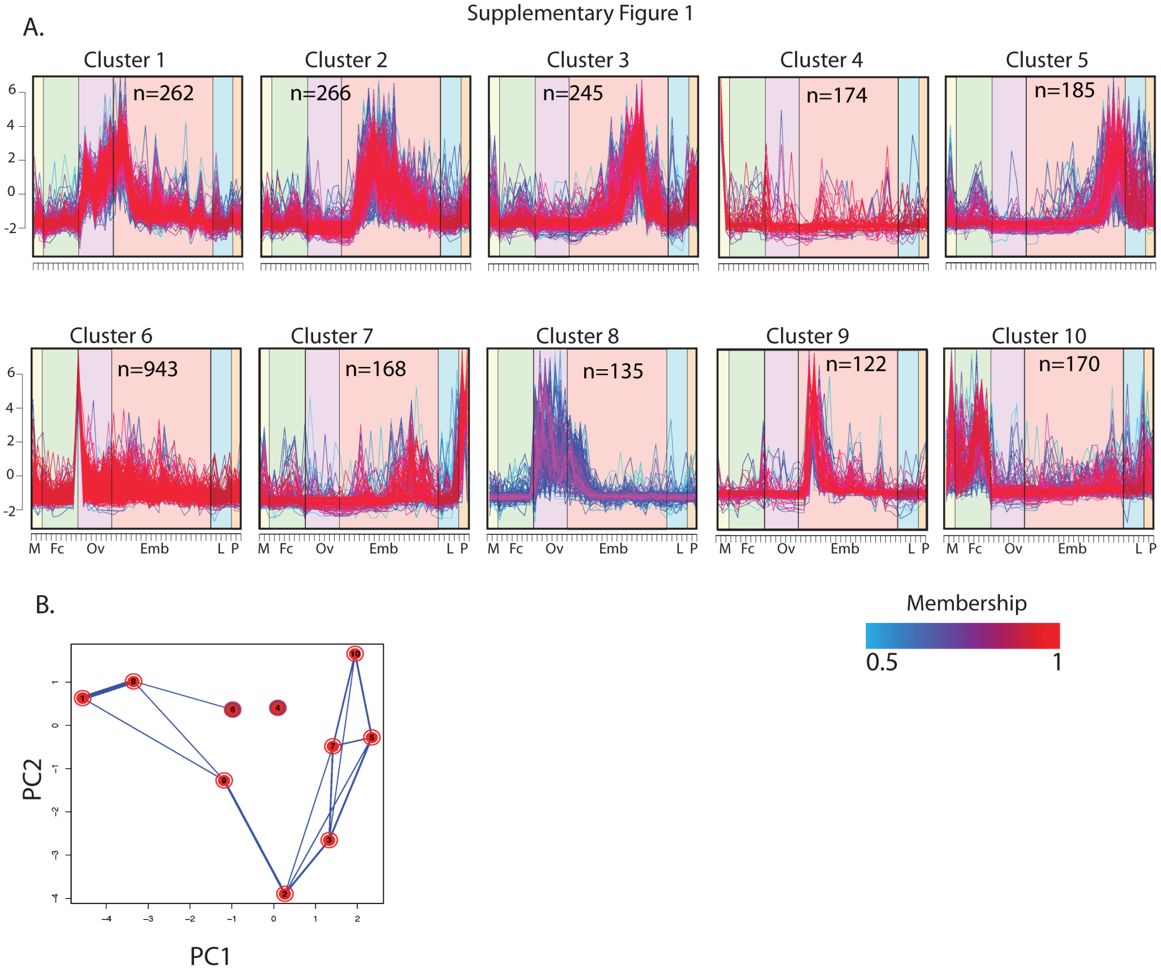

Figure S1 Soft Clustering of NTRs. 10 NTR expression profile clusters are identified through soft clustering procedure. Each NTR is assigned a line color corresponding to its membership value, with red (1) indicating high association. The major developmental groups are organized as in Figure 1B-D (A). Principal component analysis shows relationships between the 20 clusters, with thickness of the blue lines between any two clusters reflecting the fraction of genes that are shared (B, thickness of blue lines). n= the number of genes in each cluster.
