# Supplementary material for: The Developmental Transcriptome of the Mosquito Aedes aegypti, an Invasive Species and Major Arbovirus Vector
Source: G3 (Bethesda). 2013 Sep 1;3(9):1493–509. doi: 10.1534/g3.113.006742 (PMC3755910; doi:10.1534/g3.113.006742)
Supplement: Supporting Information [file supp_g3.113.006742_FigureS2.pdf]

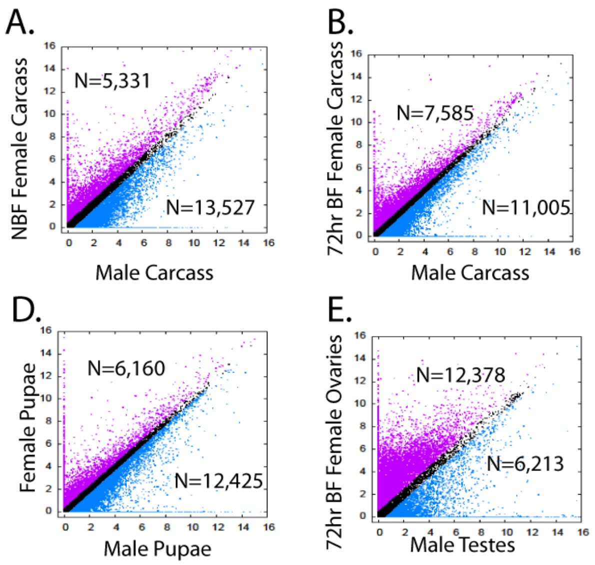

Figure S2 Scatter plots showing sex-biased gene expression. Scatter plots showing sex-bias in log(2) for AAEL genes and NTRs. 50% of the data is unbiased (black). Male biased gene expression is indicated in blue, with points falling on the X-axis showing male-specific expression. Female biased gene expression is indicated in purple, with points falling on the Y-axis showing female-specific expression. N indicates the number of genes in each category. The comparisons include non blood-fed female carcass vs. male carcass (A), female carcass 72hrs post blood feed vs. male carcass (B), non blood fed ovaries vs. testes & AG (C), female pupae vs. male pupae (D), and 72hr PBM ovaries vs. testes & AG (E). Lists of male and female sex specific genes can be located in the supplement (supplementary tables 24, 25).
